# Peer Support Systems, Psychosocial Support and Employer Attractiveness Among Healthcare Workers: A Multicenter Cross-Sectional Study in Germany

**DOI:** 10.3390/healthcare14152422

**Published:** 2026-08-06

**Authors:** Susanne Katharina Heininger, Andrea Forster, Michael Frey, Dominik Hinzmann

**Affiliations:** 1 Faculty for Applied Healthcare Sciences, Deggendorf Institute of Technology, 94469 Deggendorf, Germany; michael.frey@th-deg.de; 2 PSU-Akut e.V., 81373 Munich, Germany; andrea.forster@psu-akut.de (A.F.); d.hinzmann@tum.de (D.H.); 3 Department of Clinical Medicine, Department of Anesthesiology and Intensive Care, TUM School of Medicine and Health, Technical University of Munich, 81675 Munich, Germany

**Keywords:** peer support system, healthcare workers, psychosocial support, employer attractiveness

## Abstract

**Highlights:**

**What are the main findings?**

- Nearly half of healthcare workers (46.4%) reported experiencing at least one serious work-related event within the previous two years, underlining the importance of providing psychological support for healthcare workers.
- Peer support systems were perceived as a valuable and low-threshold resource for coping with work-related stress, and approximately one-quarter of respondents indicated that the availability of psychosocial support could influence their choice of employer.

**What are the implications of the main findings?**

- Peer support systems may strengthen perceived organizational support and improve employer attractiveness by addressing healthcare workers’ psychosocial needs following job-inherent critical incidents.
- Integrating peer support into routine organizational structures represents a feasible workforce strategy that may contribute to staff well-being, workforce stability, and sustainable healthcare delivery.

**Abstract:**

Background: Healthcare professionals are frequently exposed to serious work-related events that may adversely affect their psychological well-being, work ability, and professional retention. Peer support systems have been proposed as a low-threshold organizational approach to providing psychosocial support following such job-inherent events. This study examined the incidence of serious work-related events among healthcare workers, employees’ perceptions of an implemented peer support system, and the potential relevance of psychosocial support for employer attractiveness. Methods: A multicenter cross-sectional survey was conducted following the implementation of peer support structures in three German hospitals in 2022. A total of 295 healthcare workers completed an anonymous questionnaire assessing experiences with serious work-related events, perceptions of the peer support system, preferred support services, and the potential influence of psychosocial support on employer choice. Descriptive statistics and bivariate correlations were performed. Results: Overall, 46.4% of participants reported experiencing at least one serious work-related event within the previous two years. The peer support system was perceived as a valuable resource for supporting staff and managing psychosocial stress. Participants reported benefits not only from direct use of peer support but also from knowing that support was available when needed. Negative perceptions of the peer support system were uncommon. Approximately 27% of respondents indicated that the availability of psychosocial support could influence their choice of employer. Conclusions: Peer support systems are perceived as an acceptable and beneficial approach to addressing psychosocial stress among healthcare professionals. Beyond their supportive function, such systems may be perceived as part of employer attractiveness and a feasible organizational strategy to support workforce well-being and workforce stability in healthcare settings.

## 1. Introduction

Work-related psychological stress caused by so-called serious or adverse events can have an impact on a healthcare professional’s ability to remain in employment [1,2,3,4,5]. Serious events are understood to be emergency events and stressful events (e.g., events involving frustrated resuscitation, fatalities after accidents or events with emotionally stressful circumstances (e.g., mother dies in childbirth or violence against staff)) [6]. These examples have in common that they “[…] usually occur suddenly and unexpectedly, are (perceived to be) life-threatening for oneself or others, can lead to death, make planned action extremely difficult, cause incapacity to act and helplessness or can lead to automated behavior.” [7]. Serious events can lead to psychological trauma, which can result in acute stress reactions or even post-traumatic stress disorder [7]. As a result of serious events, staff can become second victims [8,9], which can lead to decision-making uncertainty, overdiagnosis and reduced ability to work and therefore has an impact on patient safety.

Psychosocial support in response to serious events is an interdisciplinary approach. In this study, psychosocial support is understood as an organization-based, interdisciplinary approach that provides structured emotional and practical assistance to healthcare workers following serious work-related events. It combines peer-delivered support with access to professional resources and aims to help staff cope with acute psychological stress, maintain work ability, and reduce longer-term adverse consequences. The peer support system examined here represents a low-threshold organizational approach in the sense that it is easily accessible, voluntary, and embedded into routine clinical structures without formal referral procedures, diagnostic requirements, or financial barriers. Peers are available within the workplace, contact can be initiated confidentially and at short notice, and the service is designed to complement–rather than replace–broader organizational support measures and conventional psychological or psychotherapeutic services. It is delivered by health professionals trained in crisis intervention, counseling and psychotherapy, and has been integrated into emergency response protocols across various countries. The concept is adapted to diverse sectors, including pre-service emergency medical service and humanitarian aid [10]. Psychosocial support is currently in use in only a limited number of hospitals but can serve as a crucial factor in retaining healthcare professionals [11]. Initial studies indicate that the effectiveness of peer support can be enhanced by a range of supplementary impact factors [12,13]. These include psychoeducation, validation, and the provision of relevant information, as well as strategies aimed at conserving personal resources and enabling a change in perspective. Further contributing factors are emotional containment, problem-solving, resource activation, and updating one’s understanding of the problem. In addition, fostering acceptance of change, reducing and regulating emotions, promoting a sense of connectedness, and strengthening self-efficacy all support the impact of peer support interventions. In the long term, better psychosocial support will enable healthcare workers to deal with the challenges of the profession. This can reduce staff turnover and increase staff and system retention, which can contribute to the stability of the healthcare workforce.

Systematic psychosocial support uses peers as the low-threshold first level of support [14,15]. A peer support system refers to a coordinated structure within a hospital that provides psychosocial support through trained peers, integrated with key organizational units such as management, human resources, occupational health, and quality assurance. This system includes defined processes, documentation, procedural guidelines, and communication tools. Peers are trained by sector-experienced institutions, with a focus on psychosocial stress, intervention strategies, and referral pathways into standard healthcare services. Within this framework, the peers act as guides through the psychosocial support system [7].

The objectives of a peer support system address various groups within the organization and serve multiple target populations simultaneously. These objectives include contributing to the preservation of employee health and enhancing the sense of safety among staff. In addition, peer support aims to impart coping strategies for exceptional situations and to positively influence patient safety by restoring functional stability within medical teams following critical events. Further anticipated outcomes are a more rapid return to regular work processes and quicker access to specialized support services, such as psychotherapy, which may in turn contribute to a reduction in sick leave. A peer support system is just as feasible for a small clinic as it is for large university hospitals [7]. 

Previous research has highlighted the potential value of peer support and psychosocial interventions for healthcare workers affected by serious work-related events, but there is limited evidence on how implemented peer support systems are perceived in routine clinical practice and how such systems may relate to employer attractiveness or job retention. Existing studies have mainly focused on conceptual descriptions, second-victim experiences, or specific intervention programs, with less emphasis on multicenter data from real-world hospital settings and on staff perceptions of organizational support as a possible factor in recruitment and retention. The present study seeks to address this gap by examining healthcare workers’ experiences of serious events, their perceptions of an implemented peer support system, and the perceived relevance of psychosocial support for choosing or remaining with an employer.

Job resources such as social and organizational support can buffer the negative effects of high job demands and contribute to employee well-being and retention. In this context, peer support systems may represent a relevant organizational resource in healthcare settings. Based on these results, the following research question is to be addressed in this paper: Are peer support systems perceived as a helpful organizational resource for coping with serious work-related events and as a factor that may influence healthcare professionals’ intention to remain with their employer?

The following sub-questions are to be answered:

1. What is the incidence of serious events in the participating hospitals?
2. How do employees in the target group perceive the peer support system introduced in the pilot project in terms of its effectiveness in addressing psychosocial stress at work?
3. Is a support system for dealing with serious events considered relevant to the choice of employer by the sample?

## 2. Materials and Methods

### 2.1. Aim

A pilot project entitled Practice Compass Psychosocial Prevention (duration December 2020–November 2022) was funded by the Bavarian State Ministry of Health and Care in Germany and carried out by a specialized organization for psychosocial support “PSU-Akut e.V.” in Munich. Three project hospitals, general community hospitals with a regional focus of care, were supported in (1) developing training programs for healthcare professionals on managing serious events and (2) designing and implementing a psychosocial peer support system for hospital staff. The aim of this study is to examine healthcare workers’ experiences of serious work-related events, their perceptions of an implemented peer support system, and the perceived relevance of psychosocial support for choosing or remaining with an employer in German hospital settings.

### 2.2. Design

The data collection was designed as a cross-sectional comparative study around one year after implementation of the peer support program. The questionnaire aimed to record the experienced serious events as well as the perceived support provided through psychosocial support in the form of a peer support system.

### 2.3. Data Collection

Data were collected using the online survey tool “Limesurvey” (https://www.limesurvey.org/, accessed on 29 July 2026). Eligibility was defined as employment in one of the target professional groups addressed by the pilot project (healthcare workers) and provision of informed consent; no additional formal exclusion criteria were applied beyond withdrawal of consent. The sampling strategy thus corresponds to voluntary (convenience) sampling within the project hospitals. Different approaches (emails, flyers, personal approach) were used to reach the target group with the request to participate in the survey. As multiple recruitment channels were used and participation was voluntary, the response rate could not be determined. Missing data were handled according to predefined rules (casewise exclusion for specific analyses). Various internal hospital departments were involved in the collection of the sample: Company health management, corporate communications/marketing, data protection officer, general management. Data collection in the participating hospitals took place approximately one year after the introduction of the peer support program. Because the hospitals started at different points in time, the survey was conducted between March and August 2022.

### 2.4. Questionnaires

All individuals who participated in the study were fully informed about the use of data, data protection and contact options before the study began. Informed consent has been obtained from the participants. An ethics approval was obtained from the Technical University of Munich, School of Medicine and Health, for all questionnaires (683/20S).

The questionnaire comprised 47 items with the following background variables: demographics, vocational background, perceived emotionally serious events and existing employers’ offers of support after emotionally extraordinary events (project-specific scales), inventory of life-changing events [16], workload [17], work-related behavior patterns [18]. The study focus was addressed by the following scales, developed specifically for the study: feedback to the implemented peer support system, personal use of peer support, active participation–being a peer, individual and systemic impact of the peer support system, preferred support services by employers or others.

The questionnaire used in this study was developed specifically for the pilot project and was designed to capture healthcare workers’ perceptions and experiences with the implemented peer support system. Item content was derived from existing concepts of psychosocial support after serious events and relevant literature, and was structured by an interdisciplinary team of clinicians and experts in psychosocial care to ensure conceptual coherence and practical relevance. However, the project did not include a formal psychometric evaluation of these project-specific scales, such as systematic analyses of internal consistency, factor structure, or external validation against established instruments. As a result, reliability and validity of the self-developed measures can only be regarded as preliminary.

### 2.5. Setting

Data collection took place at the end of the model project. The project focused on healthcare workers in community hospitals. All content was developed for an interdisciplinary target group consisting of physicians, nurses and surgical assistants, particularly in the areas of emergency, anesthesia and intensive care medicine. The sample size was determined by the real-world implementation context of the pilot project rather than by an a priori power calculation. All healthcare workers in the predefined target professional groups at the participating hospitals were invited to take part in the voluntary survey during the project period, and the final sample of 295 respondents reflects the number of staff who chose to participate under these conditions. The study was therefore conceived as an exploratory cross-sectional survey including all eligible and willing participants, with the aim of describing staff perceptions of the implemented peer support system rather than testing specific hypotheses based on a predetermined effect size.

### 2.6. Characteristics of Participants

The analysis was based on a total of 295 data sets. 30.2% of participants were employed at hospitals categorized as primary care providers, 12.7% at secondary care facilities, and 41.7% at tertiary care hospitals, reflecting different levels of service provision within the German hospital system.

#### 2.6.1. Age and Gender

The sample had a mean age of 43.4 years (*SD* = 11.9). Gender distribution was as follows: 70.1% female, 27.6% male, and 2.2% diverse.

#### 2.6.2. Profession and Professional Experience

The largest professional groups among the participants were trained healthcare and nursing staff (44.4%) and those categorized as “Other” (41.8%). The “Other” category included medical assistants, medical-technical professionals, psychologists, and therapists. Physicians made up 10.1% of the sample, while physicians in specialist training accounted for 3.0%. Additionally, 0.7% of the respondents were healthcare or nursing trainees. The average professional experience across the entire sample was 17.81 years (*SD* = 11.74).

#### 2.6.3. Working Hours and Area of Application

The majority of participants (64.3%) were employed full-time, working at least 38.5 h per week. A further 33.9% reported working part-time between 20 and 38 h per week, while 1.8% worked less than 20 h per week.

### 2.7. Statistical Analysis

Data analysis was conducted using descriptive statistics. The individual datasets from the model hospitals were merged, cleaned, and processed accordingly. Likert-scale items were analyzed descriptively (means, frequencies). In line with common practice in health services research, the Likert-scale items used in this study were treated as approximately continuous variables for selected correlational analyses. These analyses are intended to provide an exploratory description of associations rather than to support strong inferential or causal conclusions, and the ordinal nature of the underlying response scales is acknowledged as a methodological limitation. For validated scales, the evaluation procedures recommended by the original authors were applied where appropriate. In addition to descriptive analyses, bivariate correlations were calculated to examine relationships between selected variables. The significance level was set at α = 0.05 (two-sided). Prior to Pearson correlation analyses, approximate normality and linearity were evaluated. Pearson correlation was used to explore linear associations between variables treated as approximately continuous. Given the exploratory nature of the study, the findings should be interpreted as descriptive. All statistical analyses were performed using SPSS software, version 29.

## 3. Results

The following section presents the results related to each of the research questions.

### 3.1. What Is the Incidence of Serious Events in the Participating Hospitals?

A total of 46.4% of respondents reported having experienced at least one serious event in their workplace within the past two years. 22.7% had experienced a colleague’s suicide within their institution. It should be noted that this percentage reflects the proportion of healthcare professionals affected by a colleague’s suicide rather than the number of suicide events. A single event may affect multiple colleagues within a hospital setting. Across their entire careers, participants indicated an average of 10.01 serious events (*SD* = 15.22), with reported values ranging from 0 to 100. A Pearson correlation analysis revealed a significant positive relationship between years of professional experience and the occurrence of serious events (*r* = 0.16, *p* = 0.031, *N* = 174).

### 3.2. How Do Employees in the Target Group Perceive the Peer Support System Introduced in the Pilot Project in Terms of Its Effectiveness in Addressing Psychosocial Stress at Work?

The following results show how the peer support system introduced in the pilot project was perceived by the target group of employees as support in dealing with psychosocial stress in the workplace.

#### 3.2.1. Perceived Influence of Psychosocial Support on the Ability to Work

The findings indicate that the participants perceived psychosocial support through a peer support system as a valuable approach to caring for healthcare professionals (*M* = 4.38). Participants reported a high preference for working in a hospital with an established peer support system (*M* = 3.91) as well as indicated that a peer support system was perceived as supportive for continuing to work in their profession (*M* = 3.72). Notably, respondents also expressed a sense of reassurance and support from the mere existence of psychosocial support structures, even if they had not yet made personal use of them (*M* = 3.87). The introduction of peer support mechanisms to assist in coping with stressful events was viewed very positively (*M* = 4.64). (See Table 1).

#### 3.2.2. Perception of Potentially Positive Aspects of the Peer Support System Introduced

The following results reflect how participants perceived the potential benefits of the implemented peer support system (see Table 2). It was regarded as particularly positive that the hospital, as an employer, offers psychological support (*M* = 4.35) and actively strives to assist employees in emotionally challenging situations (*M* = 4.41). Notably, respondents agreed that the peer support system could help staff continue working more effectively after distressing events (*M* = 4.29) and potentially reduce the likelihood of sick leave (*M* = 3.56), both aspects that are highly relevant to addressing staff shortages and promoting workforce retention.

#### 3.2.3. Perception of Potentially Negative Aspects of the Peer Support System Introduced

The following results show the perception of possible negative aspects in the context of the introduction of the peer support system (see Table 3). Possible negative aspects were rated with less approval. Respondents *did not* think that colleagues who could not cope with the stresses of the profession should pursue another profession (*M* = 1.55). It also seemed to be well accepted that colleagues who work as peers within the clinic are allowed to do so during their working hours (*M* = 1.46). The workload was not rated as excessive (*M* = 1.54).

### 3.3. Is a Support System for Dealing with Serious Events Considered Relevant to the Choice of Employer by the Sample?

The following results highlight the types of support services preferred by the surveyed participants, as well as the potential influence of psychosocial support on their willingness to remain with or leave their current employer.

Across all listed services, respondents expressed consistently high levels of approval. The most favorably rated offerings included crisis intervention services for employees, particularly in situations where multiple team members are affected by a critical incident (*M* = 4.21); access to trained colleagues from the same department for confidential conversations, representing peer-based psychosocial support (*M* = 3.95); and in-person support contacts external to the workplace (*M* = 3.94) (see Table 4).

When asked specifically whether they would consider changing employers if the new employer offered psychosocial support, 27.1% of respondents indicated agreement (*M* = 2.96, *SD* = 1.38, Min = 1, Max = 5). In contrast, 26.1% stated they would not change employers for this reason.

## 4. Discussion

Peer support systems may function as a low-threshold organizational resource that enhances perceived support, strengthens resilience, and stabilizes employees’ intention to remain in the profession.

Nearly half of the participants (46.4%) reported experiencing serious events at work within the past two years. While no significant correlation was found with profession or gender, additional qualifications, such as emergency or intensive care specialization, were associated with a higher likelihood of encountering such events, likely due to working in high-acuity settings. Additionally, 22.7% had experienced a colleague’s suicide within their institution. This high proportion of respondents reporting exposure to a colleague’s suicide should be interpreted with caution, as it reflects the number of healthcare professionals who reported being sustainably affected by such an event rather than the number of suicide events. A single suicide within a healthcare organization may have a substantial impact on multiple colleagues, resulting in a comparatively high proportion of affected staff members. These findings are in line with prior research and emphasize the necessity of implementing structured, clinically informed postvention strategies [19,20].

The peer support systems introduced in the participating hospitals were widely perceived as beneficial. Respondents viewed psychosocial support not only as a valuable form of employer care but also as contributing to their perceived ability to manage job-related stress, both immediately and over the long term. Furthermore, participants described the mere availability of such support structures as having a potentially stabilizing effect, regardless of whether they actually made use of them.

Participants rated employer efforts to safeguard mental health positively, particularly in the context of emotionally challenging situations. Psychosocial support was seen as helpful for maintaining work ability and reducing absenteeism, an aspect directly relevant to workforce retention. Criticism of the peer support system was minimal. Common concerns such as whether peers should deliver support during working hours or whether psychosocial/psychological services should be provided exclusively by professionals were rarely reported. The surveyed healthcare professionals expressed the view that the employer bears responsibility for providing support in dealing with serious events, signaling a shift away from older notions that resilience is a purely individual matter. These findings are in line with international research [21,22].

The availability of psychosocial support was also linked to employer attractiveness. Preferred services included crisis interventions for groups, trained in-house peer supporters, and additional external points of contact. Notably, around one third of respondents indicated they might, or even certainly, switch employers if psychosocial support were available there, highlighting its potential role in staff retention.

This interest reflects growing strain on healthcare professionals due to the pandemic, rising economic pressure, and ongoing health system reforms. While the study was conducted in Germany, the findings point to the relevance of peer support systems in international settings, as they address psychosocial stress following critical events regardless of systemic or financial differences.

The findings support the integration of psychosocial peer support systems as a meaningful response to workforce challenges in the healthcare system. The pilot project demonstrated that such systems are accepted, valued, and perceived as effective. In some cases, access to psychosocial support even influenced career decisions.

Beyond structural reforms such as digitalization or improved work conditions, healthcare systems must also invest in approaches that help staff cope with the job-inherent emotional demands of their profession. Peer support systems represent a people-centered strategy to strengthen resilience, improve retention, and ultimately support both employees and the quality of patient care [23,24].

From a health services perspective, the results highlight the relevance of organizational-level interventions in addressing workforce shortages. While many strategies focus on structural reforms or financial incentives, our findings suggest that comparatively low-cost psychosocial interventions may contribute meaningfully to workforce stability. This aligns with current discussions on sustainable health systems, where maintaining the existing workforce is considered as critical as recruiting new staff.

The findings could have important implications for healthcare management and strategy. The fact that even the mere availability of peer support was perceived as beneficial suggests that such systems may have symbolic as well as functional value. This indicates that organizations can increase their attractiveness as employers not only through direct interventions but also by signaling a commitment to employee well-being. In times of increasing competition for healthcare professionals, this may represent a strategically relevant factor for recruitment and retention.

The recruitment and retention of qualified healthcare professionals remain critical success factors for sustaining medical and care quality. Research has identified a number of levers for improvement, including more structured opportunities for continuing professional development, better reconciliation of work and private life, political and institutional reforms, and overall improvement of working conditions. Survey data suggest that improvements in remuneration, reduced psychological stress, and more flexible working hours are essential to prevent further attrition. Fundamental reforms will be necessary to make the healthcare professions more attractive for future generations and to sustainably improve conditions for current healthcare workers [19,20,25].

In light of these developments, the relevance of the remaining personnel comes strongly to the forefront. The Bavarian State Ministry of Health and Care supports projects which aim to establish and implement psychosocial support structures for healthcare workers exposed to serious events. Numerous hospitals, hospital networks, municipal care institutions, and outpatient clinics have made use of the offered services. These include both direct psychosocial support for staff and strategic consulting for building internal support structures.

Importantly, these efforts are now being institutionally anchored: the topics of “coping with serious events” and “psychosocial stress” have been integrated into the National Competency-Based Learning Objectives Catalog for Medical Education (NKLM) [26] and into the curriculum for generalist nursing education [27]. These developments signal a systemic shift toward acknowledging the role of emotional resilience and mental health in staff retention and professional sustainability.

## 5. Limitations

This study has several limitations that should be considered when interpreting the findings. The cross-sectional design precludes causal inferences regarding the relationship between peer support systems and workforce retention. However, the study captures real-world implementation conditions and provides exploratory cross-sectional evidence on perceived benefits and associations. The voluntary nature of participation, the use of multiple recruitment channels, and the inability to determine the response rate may have introduced self-selection bias and limit the representativeness of the sample. The convenience sampling approach may have introduced selection bias and limits generalizability. The absence of pre-implementation measurements restricts the assessment of changes over time. Furthermore, the study did not capture actual peer support utilization, and the non-controlled design does not allow for causal inferences.

The use of self-reported data may introduce response bias. At the same time, perceived support and subjective evaluations are central to understanding the effectiveness of psychosocial interventions, making these data particularly meaningful for the research question. The questionnaire used in this study was developed specifically for this pilot project to capture clinicians’ perceptions of the peer support system and has not undergone formal psychometric testing. Reliability and validity of the project-specific scales must therefore be considered preliminary, and the reported findings should be interpreted as exploratory self-report results that require confirmation in future studies using rigorously validated instruments. Consequently, the findings should be interpreted with caution and may not be generalizable to all healthcare professionals in the participating hospitals or beyond.

A further methodological limitation arises from the treatment of Likert-scale items as approximately continuous variables in selected correlational analyses. While this approach is common in exploratory health services research, it does not fully reflect the ordinal nature of the underlying response categories and may introduce minor bias in the estimation of associations. Results should therefore be interpreted with caution and viewed as descriptive indications of potential relationships rather than as precise estimates or evidence of causal effects. In addition, the questionnaire consists of project-specific exploratory items rather than validated multi-item scales. Calculating summary scores or reporting only grouped constructs would imply a psychometric structure that has not yet been established. For this reason, we presented the individual item responses to ensure transparency and enable future studies to compare or build upon these findings when developing and validating standardized instruments.

The study was conducted in three publicly funded hospitals in Bavaria, Germany. While this may limit generalizability to other healthcare systems or institutional contexts, the inclusion of hospitals across different levels of care strengthens the applicability of the findings within comparable clinical settings.

Data collection took place during the later phase of the COVID-19 pandemic (Q2/Q3 2022). Although pandemic-related factors may have influenced response patterns, this context also reflects a period of heightened psychosocial demand, thereby increasing the relevance and timeliness of the findings.

The present study was primarily designed as an exploratory investigation to describe healthcare workers’ perceptions of peer support systems rather than to identify predictors of employer attractiveness. Future studies with larger samples should therefore examine whether demographic and professional characteristics, such as age, profession, years of professional experience, or employment status, are associated with the perceived relevance of psychosocial support for employer attractiveness and workforce retention. Such analyses may help to identify subgroups that particularly benefit from organizational psychosocial support and to tailor implementation strategies accordingly.

Overall, the study provides empirically grounded insights into the perceived value of peer support systems in healthcare. Future longitudinal and intervention-based research is needed to establish causal pathways and to assess long-term effects on workforce retention.

## 6. Conclusions

The article highlights that, in the future, healthcare organizations will need to move beyond viewing personnel solely as a cost factor [19]. For leadership in healthcare, safeguarding mental health and fostering resilience in the face of serious events will become essential responsibilities. Peer support and psychosocial interventions represent one vital component of such a supportive infrastructure [21,22,28,29]. Given the cross-sectional and exploratory design, our findings should be understood as reflecting perceived benefits and potential implications rather than causal effects on workforce retention or organizational outcomes. Future longitudinal and interventional studies are needed to clarify whether and how peer support systems influence staff retention and other organizational indicators over time.

Peer support systems represent a feasible, scalable, and well-accepted approach to addressing psychosocial stress among healthcare professionals. Beyond their direct supportive function, they may enhance perceived organizational commitment and contribute to workforce retention. In light of ongoing workforce shortages, integrating psychosocial support into routine organizational structures should be considered a strategic priority for healthcare systems. From an organizational perspective, the implementation of peer support systems requires clear structures, defined responsibilities, and integration into existing strategies. Future evaluations should focus on longitudinal outcomes and implementation processes to assess their sustained impact on healthcare organizations and workforce stability.

## Figures and Tables

**Table 1:** Perceptions of the peer support system introduced.

Item	*N*	*M* (Agreement on a 5-Point-Likert-Scale)	*SD*
I think a peer support system is a good way of caring for staff.	238	4.38	0.95
I prefer to work in a clinic where there is a peer support system for me.	237	3.91	1.27
Just knowing that there is a peer support system for me helps me, even if I do not/do not yet use it myself.	239	3.87	1.25
I think I can work better with a peer support system in the background.	238	3.64	1.25
I think a peer support system helps me to continue working in my profession.	239	3.72	1.23
I think it was a good idea to introduce a peer support system in our clinic to provide support after stressful events.	241	4.64	0.77

*Note*. The assessments were made on a 5-point Likert scale from 1 = strongly disagree to 5 = strongly agree. [Higher values correspond to higher agreement].

**Table 2:** Perception of positive aspects of the implemented peer support system.

Item	*N*	*M* (Agreement on a 5-Point-Likert-Scale)	*SD*
I believe that the peer support system can help people to avoid having to take sick leave after stressful events.	240	3.56	1.26
I believe that the peer support system can help people to continue working better after stressful events.	237	4.29	1.05
I think it is good that our clinic makes an effort to support employees even in stressful situations.	237	4.41	0.99
I tell friends/acquaintances/family about our new peer support system.	237	2.88	1.47
I have heard from other teams that the peer support system is well received.	230	3.05	1.44
My leader supports the new peer support system.	234	3.84	1.12
I feel that I can now speak up if a situation at work is bothering me.	238	3.46	1.43
I think it is good that the clinic offers mental health services.	240	4.35	0.96
I have the feeling that our clinic also cares about my mental health.	238	3.19	1.34
The atmosphere in the team has already improved.	225	2.52	1.17
I think it is good for the working atmosphere that there is now someone to talk to after stressful events.	241	4.2	1.07
I believe that it will help me in the future to have a contact person after stressful events.	241	4.07	1.12
I now know a contact person if I feel the need to talk to someone after a stressful event.	242	3.86	1.46

*Note*. The assessments were made on a 5-point Likert scale from 1 = strongly disagree to 5 = strongly agree. [Higher values correspond to higher agreement].

**Table 3:** Perception of negative aspects of the implemented peer support system.

Item	*N*	*M* (Agreement on a 5-Point-Likert-Scale)	*SD*
I think that psychologists or therapists should talk to colleagues who feel stressed after serious events.	235	2.43	1.30
I believe that this peer support system is not enough to absorb the stress after critical incidents.	234	3.06	1.22
I think that colleagues who cannot cope with the stress should pursue a different profession.	233	1.55	0.96
I think that this peer support system will only make more work for everyone in the future.	231	1.54	1.02
I think it is unfair that colleagues are now allowed to take time during their working hours for peer discussions.	232	1.46	1.02
My leader thinks the new peer support system is superfluous.	224	1.83	1.83
In my team, everyone is still expected to deal with stressful experiences themselves.	233	2.18	1.30
My team never actually talks about the fact that experiences at work can be stressful.	232	2.28	1.32
No one in my team wanted to become a peer.	215	2.63	1.42
The introduction of the peer support system made a lot of work for us.	212	2.3	1.18

*Note*. The assessments were made on a 5-point Likert scale from 1 = strongly disagree to 5 = strongly agree. [Higher values correspond to higher agreement].

**Table 4:** Preferred support services.

Item	*N*	*M* (Agreement on a 5-Point-Likert-Scale)	*SD*
Crisis intervention services for employees (if several colleagues are affected by a serious event)	203	4.21	1.13
Trained colleagues from my department in my clinic for confidential discussions (=peer support system)	205	3.95	1.26
Structured peer counseling/psychosocial counseling	203	3.89	1.21
Seminars on promoting resilience and self-protection	204	3.9	1.31
24/7 telephone hotline for exceptional emotional stress	202	3.32	1.40
Telephone contacts outside my workplace	203	3.12	1.40
Physical contact partners outside my workplace	203	3.94	1.22
Conflict management	201	3.8	1.23
Offers for conducting conversations, communication training	203	3.69	1.34
Fallback level and access to a support network for individual cases	199	3.87	1.27

*Note*. The assessments were made on a 5-point Likert scale from 1 = strongly disagree to 5 = strongly agree. [Higher values correspond to higher agreement].

## Data Availability

The data used in this study contain potentially identifiable information from healthcare professionals working in participating hospitals. Although all analyses were performed using anonymized datasets, the underlying raw data cannot be made publicly available due to ethical and data protection requirements and the conditions of the ethics approval. The data are therefore available from the corresponding author upon reasonable request, provided that the request complies with applicable ethical and legal requirements.

## Abbreviation

The following abbreviation is used in this manuscript:

PSU	Psychosocial support (German: Psychosoziale Unterstützung)
